# Characteristics, Mortality, and Clinical Outcomes of Hospitalized Patients with COVID-19 and Diabetes: A Reference Single-Center Cohort Study from Poland

**DOI:** 10.1155/2023/8700302

**Published:** 2023-02-16

**Authors:** Michał Kania, Konrad Mazur, Michał Terlecki, Bartłomiej Matejko, Jerzy Hohendorff, Zlata Chaykivska, Mateusz Fiema, Marianna Kopka, Małgorzata Kostrzycka, Magdalena Wilk, Tomasz Klupa, Przemysław Witek, Barbara Katra, Marek Klocek, Marek Rajzer, Maciej T. Malecki

**Affiliations:** ^1^Department of Metabolic Diseases and Diabetology, Jagiellonian University Medical College, Krakow, Poland; ^2^University Hospital in Krakow, Krakow, Poland; ^3^Department of Cardiology, Interventional Electrocardiology and Arterial Hypertension, Jagiellonian University Medical College, Krakow, Poland

## Abstract

**Background:**

Diabetes is a risk factor for a severe course of COVID-19. We evaluated the characteristics and risk factors associated with undesirable outcomes in diabetic patients (DPs) hospitalized due to COVID-19.

**Materials and Methods:**

The data analysis of patients admitted between March 6, 2020, and May 31, 2021, to the University Hospital in Krakow (Poland), a reference center for COVID-19, was performed. The data were gathered from their medical records.

**Results:**

A total number of 5191 patients were included, of which 2348 (45.2%) were women. The patients were at the median age of 64 (IQR: 51–74) years, and 1364 (26.3%) were DPs. DPs, compared to nondiabetics, were older (median age: 70 years, IQR: 62–77 vs. 62, IQR: 47–72, and *p* < 0.001) and had a similar gender distribution. The DP group had a higher mortality rate (26.2% vs. 15.7%, *p* < 0.001) and longer hospital stays (median: 15 days, IQR: 10–24 vs. 13, IQR: 9–20, and *p* < 0.001). DPs were admitted to the ICU more frequently (15.7% vs. 11.0%, *p* < 0.001) and required mechanical ventilation more often (15.5% vs. 11.3%, *p* < 0.001). In a multivariate logistic regression, factors associated with a higher risk of death were age >65 years, glycaemia >10 mmol/L, CRP and D-dimer level, prehospital insulin and loop diuretic use, presence of heart failure, and chronic kidney disease. Factors contributing to lower mortality were in-hospital use of statin, thiazide diuretic, and calcium channel blocker.

**Conclusion:**

In this large COVID-19 cohort, DPs constituted more than a quarter of hospitalized patients. The risk of death and other outcomes compared to nondiabetics was higher in this group. We identified a number of clinical, laboratory, and therapeutic variables associated with the risk of hospital death in DPs.

## 1. Introduction

COVID-19, a disease caused by the SARS-CoV-2 virus, characterized by a high mortality rate, particularly in the elderly population, has spread rapidly around the world, which resulted in a declaration of the global pandemic by the WHO on 11^th^ March, 2020 [1]. The first case of COVID-19 in Poland was identified on March 3^rd^, 2020. After that, the first wave of the pandemic occurred, which triggered the first national lockdown that lasted approximately until the end of June 2021.

According to the statistics concerning COVID-19, from the European Centre for Disease Prevention and Control, Poland has been one of the most severely affected countries of the world [2]. Until the end of January 2022 in Poland, SARS-CoV-2 infected over 4,850,000 people with a total number of deaths of approximately 105,000 [3]. According to official data from the Polish government, a total of 68,505 excess deaths were recorded in 2020, which accounts for a 16% increase in total mortality compared to the period 2017–2019 [4].

The COVID-19 pandemic deeply affected the healthcare system in Poland. Due to an increasing number of cases of the disease, the Polish Ministry of Health decided to form a system of hospitals dedicated solely to serve as multispeciality reference centers for COVID-19 patients [5, 6]. Additionally, temporary hospitals were established and numerous regional hospitals were converted to play a supportive role [7].

The most common reported comorbidities of COVID-19 were arterial hypertension, type 2 diabetes (T2DM), cardiovascular disorders (CVDs), and respiratory system diseases [8]. Many reports from different populations have been pointing to diabetes as a strong and independent risk factor for a severe course and increased mortality in COVID-19 [9, 10]. For example, diabetic patients (DPs) constituted up to 20% of the hospitalized patients with COVID-19 [8, 11]. Furthermore, COVID-19 patients with pre-existing diabetes showed a mortality risk two to three times higher than nondiabetics [8, 12, 13].

Retrospective observational data showed that better glycemic control in hospitalized DPs with COVID-19 was associated with a reduced in-hospital mortality compared to patients with poorly controlled glycaemia [14]. The impact of glucose-lowering drugs on the course of SARS-CoV-2 infection is unclear and remains under investigation. Several observational retrospective studies in DPs with COVID-19 suggested a beneficial effect of using metformin [15, 16], while another report found that insulin therapy in patients with T2DM was associated with increased mortality [17]; although, these observations should be treated with caution due to methodological limitations.

Taking into account the number of DPs infected with SARS-CoV-2 and severe clinical course of COVID-19 in patients with diabetes, it is important to continue collecting and reporting data from hospital cohorts of this group of patients.

The purpose of this study was to assess the clinical characteristics and risk factors associated with unfavorable outcomes in DPs hospitalized due to COVID-19.

## 2. Methods

This report covers medical records of 5191 consecutive patients admitted between March 6, 2020, and May 31, 2021, to the University Hospital (UH) in Krakow. Since March 2020, UH has temporarily been transformed into the regional COVID-19 center. In October 2020, after the Ministry of Health had established 16 coordinating hospitals overseeing the local network of COVID-19 medical centers, UH in Krakow became the coordinating unit for patients with SARS-CoV-2 infection in Lesser Poland. This unit was responsible for the hospitalization of patients with COVID-19 requiring specialized treatment, for example, due to myocardial infarction (MI), stroke, pregnancy, psychiatric disorders, or acute respiratory failure requiring mechanical ventilation in an intensive care unit (ICU) [18, 19]. Diagnosis with COVID-19 was made according to the WHO and Polish guidelines with the use of the RT-PCR method [20, 21]. The COVID-19 treatment algorithm was based on constantly updated recommendations of the Polish Association of Epidemiologists and Infectiologists [20].

Demographic and clinical data were extracted from the hospital's digital medical records. The first dataset, concerning 1729 patients, was developed, analyzed, and published in another report [19]. The database of all the patients included information on patients' age, sex, date of admission, date of discharge or death, admission to the ICU, and use of mechanical ventilation. The characteristics also involved comorbidities (previous diagnosis of diabetes, arterial hypertension, heart failure (HF), history of MI or stroke, ischemic heart disease, atrial fibrillation (AF), chronic kidney disease (CKD), and chronic obstructive pulmonary disease (COPD) and in-hospital treatment (angiotensin-converting enzyme inhibitors (ACEIs), angiotensin II receptor blockers (ARBs), beta-blockers (BBs), vitamin K antagonists (VKAs), new oral anticoagulants (NOACs), calcium channel blockers (CCBs), remdesivir, and low molecular weight heparin (LMWH). CVD and cardiovascular risk factors were identified on the basis of medical history of prehospital diagnosis and/or treatment and defined in accordance to the current European Society of Cardiology guidelines [22]. Similarly, other chronic comorbidities, COPD and CKD, were also recognized based on earlier diagnosis available in the medical records. Baseline clinical parameters at admission were extracted. They included heart rate, blood pressure, oxygen saturation, respiratory rate, as well as laboratory results (C-reactive protein (CRP), D-dimer, white blood count (WBC), and plasma glucose).

With further development of the database, the type of diabetes and information on prehospital diabetes treatment (use of metformin, sulfonylurea (SU), SGLT2 inhibitor, GLP-1R agonist, DPP4 inhibitor, and insulin) as well as the level of glycated hemoglobin (HbA1c) were added to the DP records. The list of collected characteristics is shown in Tables 1 and 2.

The main analyzed outcome of the study was in-hospital death from any cause. Additionally, we analyzed secondary end points such as length of stay until death or discharge, ICU admission, and mechanical ventilation requirement. These outcomes were evaluated and compared between the DP and the nondiabetics group. We also searched for independent risk factors for in-hospital death in DPs by building a multivariate logistic model including the following variables: age >65 years, glycaemia on admission greater than 10 mmol/L, CRP, D-dimer and WBC level on admission, HF, coronary artery disease, atrial fibrillation, CKD, COPD, history of MI, prehospital use of metformin and insulin, in-hospital use of ACEI/ARB, BB, CCB, loop diuretics, thiazide diuretics, and statin. Another model was built to identify the independent risk factors for the prediction of admission to the ICU including the following variables: gender, age, glycaemia on admission greater than 10 mmol/L, CRP, D-dimer and WBC level on admission, atrial fibrillation, in-hospital use of antiplatelet drugs, BB, loop diuretics, and thiazide diuretics.

This study was entirely observational with no deviation from standard clinical care. The authors, all employees of the University Hospital, had been granted a special permission from the Krakow University Hospital authorities to access the data. The study was approved by the Jagiellonian University Ethics Committee, decision number 1072.6120.278.2020.

### 2.1. Statistical Analysis

All statistical analyses were made using the *R* software, version 4.1.1. The normality of the continuous variable distribution was assessed using the Shapiro–Wilk test. Differences between groups were analyzed with Student's *t*-test or nonparametric tests (the Mann–Whitney *U*-test), when appropriate. Continuous variables were presented as arithmetic means (*x̄*) ± standard deviations (SD) or median (interquartile range; IQR) when the data were not normally distributed. The distribution of categorical variables was shown by counts and percentages. Statistical testing was completed to compare the categorical variables using the independent samples chi-square test or Fisher's exact test when appropriate. To search for factors associated with hospital death and admission to the ICU of DP, we performed univariate logistic regression using the variables listed in Table 1 and marked with an asterisk. Then, we performed multivariate logistic regression, including predictors that were significant in univariate logistic regression and did not cause deletion of more than 350 cases, an arbitrary chosen number corresponding to less than 30% of lost data (“age in class” over “age” and “glucose over 10 mmol/L” or “glucose” were chosen; Tables 3 and 4). The multicollinearity assumption was fulfilled in the final models. The Nagelkerke's index was used as an equivalent of the coefficient of determination, R2. The strength of the association was measured by the odds ratio (OR) and the 95% confidence intervals (CI). Statistical inference was based on the criterion *p* < 0.05.

## 3. Results

Between March 6, 2020, and May 31, 2021, 5191 patients were hospitalized due to COVID-19 in the UH in Krakow. Of this number, 2348 (45.2%) were women and 2843 (54.8%) were men, and 2409 (46.4%) patients were older than 65 years, with the mean age of 61.98 ± 16.66 years. We identified 1364 (26.3%) DPs with a diagnosis established prior to hospitalization.

DPs, compared to nondiabetics, were older (median age: 70 years, IQR: 62–77 vs. 62, 47–72, and *p* < 0.001), with a similar gender distribution (females: 44.4%, *n* = 606 vs. 45.5%, *n* = 1742, and *p* = 0.506), a higher BMI (30.0, IQR: 26.4–34.6 vs. 27.8, IQR: 24.7–31.4, and *p* < 0.001), and a higher prevalence of CVD diseases (87.6%, *n* = 2295 vs. 55.1%, *n* = 2109, and *p* < 0.001).

The DP group was characterized by a higher mortality (26.2%, *n* = 358 vs. 15.7%, *n* = 599, and *p* < 0.001), longer hospital stays (median: 15 days, IQR: 10–24 vs. 13, 9–20, and *p* < 0.001), more frequent admission to the ICU (15.7%, *n* = 214 vs. 11%, *n* = 420, and *p* < 0.001), and mechanical ventilation requirement (15.5%, *n* = 211 vs. 11.3%, *n* = 432, and *p* < 0.001). Data on the main clinical outcomes in DP and nondiabetics are summarized in Table 3.

The majority of DPs had T2DM (93.9%), and the data concerning weight were available for 583 patients, from which 494 (84.7%) were overweight or obese. The most common comorbidities were arterial hypertension (82.9%) followed by hyperlipidemia (33.7%), coronary artery disease (28.9%), atrial fibrillation (18.5%), HF (17.2%), previous MI (16.7%) and stroke (11.5%), CKD (14.8%), and COPD (8.4%). DPs with COVID-19 who died during hospitalization were older and had a higher prevalence of pre-existing comorbidities as shown in Table 1. This table presents the basic characteristics of DP together with their prehospital and hospital treatment in survivors and nonsurvivors.

Prehospital use of metformin was more frequent in the survivor group than in nonsurvivors, while prehospital insulin usage was more frequent in nonsurvivors. The frequency of in-hospital use of metformin, SU, SGLT2 inhibitors, ACEI/ARB, BB, VKA/NOAC, statin, CCB, and thiazide diuretics was higher in survivors than that in nonsurvivors. Insulin, loop diuretics, dexamethasone, and LMWH were used more commonly in the nonsurvivor group. There was no difference in the frequency of remdesivir used between the survivors and nonsurvivors groups. Metformin was discontinued on hospital admission in 283 DP (40.6%) that had previously used this medication. This subgroup, when compared with those treated with metformin during the hospital stay, was characterized by a higher mortality (35% vs. 5.5%, *p* < 0.001), more frequent admission to the ICU (19.8% vs. 6.8%, *p* < 0.001), and mechanical ventilation requirement (21.6% vs. 6.5%, *p* < 0.001). Patients in whom metformin was discontinued on admission had higher CRP, D-dimer, and blood glucose and lower oxygen saturation (Supplementary Table S1).

Patients who died during hospitalization, compared to those who recovered, had lower blood pressure (both systolic and diastolic), oxygen saturation, and a higher respiratory rate upon admission. Inflammatory markers (CRP, WBC), D-dimer, creatine, and blood glucose at admission were higher in the nonsurvivor group. The results of all these comparisons are also presented in Table 1.

A multivariate logistic model for the predictors of mortality showed that age of over 65 years, higher CRP or D-dimer or glycaemia greater than 10 mmol/L upon admission, history of HF and CKD, prehospital insulin use, and in-hospital loop diuretics use were associated with a higher death rate, while the in-hospital use of statin, thiazide diuretic, and CCB were associated with lower mortality (Table 2).

In a multivariate logistic model for the predictors of stay in intensive care unit of DPs, a higher CRP level, glycaemia over 10 mmol/L upon admission, and in-hospital use of loop diuretics were associated with a higher risk of stay in the ICU, whereas older age and the in-hospital use of thiazide diuretics were related with a lower risk of stay in ICU (Table 4).

## 4. Discussion

In this article, we present the data from a large retrospective analysis involving DPs with COVID-19 hospitalized in the UH in Krakow during the first three waves of the pandemic in Poland. We provided the clinical characteristics of DPs, compared their important selected outcomes with nondiabetics, and identified some factors associated with prognosis in diabetic cohort. The scientific significance of our findings and the potential causes of the associations identified are discussed.

This is the first large cohort study of DPs hospitalized with COVID-19 in Poland. Several research groups in this country have investigated the general population of hospitalized patients with COVID-19, with some of them exploring risk factors associated with unfavorable outcomes, including comorbidities, demographic characteristics, and laboratory findings on admission [23–25]; however, none of these reports focused on patients affected by diabetes. First, our real-world evidence report showed, similarly to previous publications, the clinical significance of diabetes, depicted in a large proportion of DP among the hospitalized patients with COVID-19. In the UH in Krakow, the largest adult hospital in Lesser Poland, DPs constituted up to a fourth of the hospitalized patients with COVID-19. This report provides data on the proportion of types of diabetes among hospitalized COVID-19 DP, showing more than 90% of the patients having T2DM. The prevalence of type 1 diabetes, GDM, and other types of disease was very low.

Diabetes was a risk factor for a severe course of COVID-19, meaning the necessity of admission to the ICU, mechanical ventilation, and longer hospital stay. However, it should be noted that the prevalence of cardiovascular and renal comorbidities was higher in our DP cohort than in nondiabetic patients. Our findings are consistent with an already published data [11, 12, 26]. Several systematic reviews and meta-analyses covered the impact of diabetes on COVID-19 outcomes [8, 11, 12, 27–30]. They revealed an approximately two- to three-fold increase in mortality due to COVID-19 for DPs compared with people without diabetes [11, 12]. Diabetes was also associated with a more severe course and progression of COVID-19 in the affected patients [9, 11] and that observation is confirmed in our study. In this report, the relative mortality risk associated with diabetes was lower than in some earlier meta-analyses [11, 12, 27, 29]. However, a number of these studies included only the source material from 2020, while the data in this study also encompass a part of 2021. Second, some meta-analyses regarding mortality were limited only to Asian patients, potentially skewing the results [11, 29]. In a recent review that included relatively more studies from the United States and Europe, the results were more similar to ours, with a higher risk of in-hospital mortality in Asian studies compared to non-Asian ones in sensitivity analyses [30]. Finally, our data come from the tertiary university center with the availability of a diabetology department and a team of specialists in this field performing consultations.

Arterial hypertension was the most common cardiovascular comorbidity in our DP cohort, followed by coronary artery disease, atrial fibrillation, history of MI and stroke, CKD, and COPD. When considered individually, some of these cardiovascular comorbidities appeared to be a risk factor of poor prognosis of COVID-19; however, when analyzed together with other patients' characteristics, their association could not have been confirmed. After adjustment in the multivariate model, only HF and CKD remained significant. Some earlier studies revealed similar observations [19, 31–35].

The glucose level on admission was a variable identified as an independent risk factor for in-hospital mortality. This association between glycaemia in hospitalized patients with COVID-19 and the risk of death was previously reported in many individual studies [14, 26, 36–38] and summarized in a recent meta-analysis [39]. However, glycaemia on admission could have been influenced by other factors, including inflammation, severity of infection, previous glycemic control, and stress, making this parameter difficult to interpret. Interestingly, such a relationship did not exist for the HbA1c level in our study. However, for most DPs, the HbA1c level was not measured; so, this analysis was probably influenced by that fact. In an earlier study, chronic hyperglycemia, expressed by the HbA1c level as its surrogate, was associated with a higher susceptibility to severe pneumonia [40]. However, the lack of such association, as observed in our and multiple previous studies in COVID-19 [26, 36], suggests a more complex relationship between chronic hyperglycemia and SARS-CoV-2 infection. Previous studies have shown that preadmission insulin treatment was associated with a higher risk of COVID-19-related death [17, 36], while preadmission metformin treatment lowered that risk [15, 16, 27]. We believe that the mode of hypoglycemic treatment should be considered merely as a marker of a longer duration of diabetes, more advanced complications, and usually higher prevalence of comorbidities [41]. One subanalysis of our study revealed that metformin was discontinued in nearly half of the DPs who were using it before admission. These patients were characterized by more advanced stage of COVID-19 infection on admission and a higher ICU admission rate, more frequent initiation of mechanical ventilation, and finally, higher mortality. Although these data were as expected, to date, there were no reports on this specific subgroup of DPs. The discontinuation of metformin in severe COVID-19 was recommended by numerous guidelines since the beginning of the pandemic [42, 43]. Since the evidence for safety of metformin use in the selected patients emerged, it has been recommended to sustain the metformin therapy in patients with less severe COVID-19 [42].

It is noteworthy that one meta-analysis searched for specific characteristics and phenotypes of DPs linked with a higher rate of in-hospital death [27]. The authors identified male sex, age >65, pre-existing comorbidities, including cardiovascular diseases, CKD, COPD, prehospital insulin use, and blood glucose on admission ≥11 mmol/l to be associated with the investigated outcome. Interestingly, contrary to the earlier research on general population and DPs with COVID-19, we did not find male sex as the risk factor. One may consider a possibility that women affected with diabetes, unlike in the general population [44], might have a similar risk of death due to COVID-19 as males. However, further research is necessary.

To search for the association of use of certain drugs with the outcomes of COVID-19, both examined in this study, we performed many association analyses. All of them should be treated with caution, as they are prone to all manner of biases typical for retrospective analysis based on medical records. Thus, no causative conclusions should be drawn.

The use of statin was associated with lower mortality in patients with diabetes in this study. Data concerning the role played by statins in the course of COVID-19 are conflicting, with studies reporting both lower [45] and higher [26] risk of in-hospital death. Contrary to some earlier reports [19, 46], the use of ACEI/ARB and BB was not associated with a lower risk of death from COVID-19; however, these studies did not investigate selected DP cohorts. The data on the association of other antihypertensive drugs on COVID-19-related mortality are limited. In a large retrospective cohort study, the use of any diuretic was not associated with higher mortality after adjustments for other factors [47]. In our study, loop diuretics were associated with higher mortality, while thiazide diuretics and calcium channel blockers were associated with lower mortality. This probably results from the confounding effect of more frequent use of loop diuretics in the patients with a previous diagnosis of HF. In addition, loop diuretics are also used in acute states associated with severe COVID-19, such as acute kidney injury or exacerbation of HF. On the contrary, thiazide diuretics could have been discontinued in the case of COVID-19 progression. The data concerning the role of thiazide diuretics and CCB in COVID-19 are ambiguous, with very limited number of studies investigating their association with clinical outcomes [48].

Finally, our data suggest that in-hospital mortality was higher in patients with a higher level of inflammatory markers and worse clinical symptoms (tachycardia, hypoxia, and low blood pressure) during the examination on admission. These parameters should be treated as markers of COVID-19 severity, respiratory distress, and systemic inflammation. This is in line with the already published data that revealed an association between some clinical on-admission symptoms and higher mortality in COVID-19 DP [26].

The prevalence of hyperlipidemia was underestimated in this material in comparison to similar cohorts [49]; additionally, more than 30% of the records were lacking these data. Thus, hyperlipidemia was not included into the multivariate model in spite of the surprising protective result in univariate analysis for COVID-19-related death. Previous data revealed that it had either neutral [50] or negative impact [51, 52] on survival in COVID-19 patients. One may hypothesize that it constitutes a marker of a better nutritional status that could play a role in assessing the mortality risk, particularly among the elderly population [53]. Overweight and obesity were previously identified as risk factors for severe COVID-19, regardless of diabetes diagnosis [36, 54, 55]. Interestingly, the relationship between abnormal BMI and COVID-19 risk in geriatric population is more complex, as obesity was not associated with higher hospital mortality; however, such a relationship was proven for underweight and malnutrition in this population [53, 56, 57]. In our cohort, where most of the patients were overweight or obese, we found no evidence of association between higher BMI and the examined COVID-19 outcomes. It is noteworthy that we lack data on BMI in about two-thirds of the patients and the fact that the study was probably underpowered to assess such associations.

This study is characterized by limitations typical for retrospective observational analysis. First, due to the nature of this study, our report cannot prove any causal relationship, for example, related to any of the analyzed drugs. This, for example, applies to the reported association between the prehospital insulin and in-hospital mortality. Additionally, our cohort was not clinically homogeneous, as the data analysis covered the 15-month period between the first cases of COVID-19 in the region in March 2020 and May 2021, when vaccinations against SARS-CoV-2 were widely available. Furthermore, our results could have been hampered by missing data, particularly regarding BMI, HbA1c level, hyperlipidemia, or oncological history. As a vast majority of study participants had T2DM, our results should be interpreted with caution in populations with other types of diabetes (T1DM, MODY, and so on). Last, prehospital diabetes treatment and in-hospital treatment of nondiabetic conditions were used for analysis. We did not include in-hospital hypoglycemic treatment for the association analysis; as according to the guidelines, it is recommended to switch patients with severe infections requiring hospitalization to insulin [58]. For the remaining drugs, only data from the hospitalization period were systematically collected in the database. In spite of these shortcomings, our study has been the first and most thorough investigation from Poland to date, covering a large cohort of DP with COVID-19 and diabetes requiring hospitalization. This is also one of the largest reported one-center DP cohorts reported so far in Europe.

In summary, in this large COVID-19 hospital cohort, DP constituted more than a fourth of hospitalized patients. Their risk of death and other important clinical outcomes were significantly higher compared to those of nondiabetics. We identified a number of clinical, laboratory, and therapeutic variables associated with the risk of hospital death in DP. Some of them constituted a confirmation of earlier reports; however, some other observations, for example, related to equal risk in both genders, are new and warrant further research. The results reported here may be useful for developing strategies in DP for future similar health crises. This knowledge should also allow a more accurate quantification of the risk of in-hospital mortality and other unfavorable in-hospital outcomes to guide treatment strategies.

## Figures and Tables

**Table 1:** Comparison of basic characteristics and clinical outcomes of DP and nondiabetic patients.

Characteristics	All	Diabetes	Nondiabetics	*P* value	OR (CI 95% adjusted for age and gender)	*P* value
Number	5191	1364	3827			
Age (years)	64 (51–74)	70 (62–77)	62 (47–72)	<0.001	NA	NA
Age class >65 years (*N* (%))^*∗*^	2409 (46.4%)	869 (63.7%)	1540 (40.3%)	<0.001	NA	NA
Female (*n* (%))	2348 (45.2%)	606 (44.4%)	1742 (45.5%)	0.506	NA	NA
BMI (kg/m^2^)^*∗*^	28.3 (25.2–32.1)	30.0 (26.4–34.6)	27.8 (24.7–31.4)	<0.001	NA	NA
CVD (*n* (%))	3304 (63.6%)	1195 (87.6%)	2109 (55.1%)	<0.001	NA	NA

*Endpoints*						
In-hospital death (yes (%))	957 (18.4%)	358 (26.2%)	599 (15.7%)	<0.001	1.22 (1.04–1.43)	0.016
Mechanical ventilation (yes (%))	643 (12.4%)	211 (15.5%)	432 (11.3%)	<0.001	1.33 (1.11–1.60)	0.002
Admission to an ICU (*n* (%))	634 (12.2%)	214 (15.7%)	420 (11%)	<0.001	1.44 (1.20–1.73)	<0.001
Length of hospital stay (days: median (IQR))	14 (9–21)	15 (10–24)	13 (9–20)	<0.001	1.01 (0.996–1.019)	0.226

Data are presented as median (Q1–Q3) or *N* (%); BMI, body mass index; CVD, cardiovascular disease: ischemic heart disease including the history of myocardial infarction, and cerebrovascular disease including the history of stroke, heart failure, arterial hypertension, atrial fibrillation; ICU, intensive care unit. ^*∗*^Data available, *N* = 2403.

**Table 2:** Comparison of DP patients according to the survival status together with univariate logistic regression results.

Clinical feature	Available data	All (*N* = 1364)^	In-hospital death			Univariate logistic regression	
			No (*N* = 1003)	Yes (*N* = 361)	*P* value	OR (95% CI)	*P* value
Sex (female)^*∗*^	1364	606 (44.4%)	456/1003 (45.5%)	149/361 (41.3%)	0.318	0.84 (0.66–1.07)	0.169
Age (years)^*∗*^	1364	70 (62–77)	68 (61–76)	74 (67–81)	<0.001	1.05 (1.04–1.06)	<0.001
Age class >65 years (*N* (%))^*∗*^	1364	869 (63.7%)	586/1003 (58.4%)	283/361 (78.4%)	<0.001	2.58 (1.96–3.43)	<0.001
BMI (kg/m^2^)^*∗*^	582	29.9 (26.4–34.6)	29.8 (26.5–34.6)	30.00 (25.9–34.3)	0.461	1.00 (0.97–1.03)	0.948
BMI >25 kg/m^2^ (*N* (%))^*∗*^	582	493 (84.7%)	369/428 (86.2%)	124/155 (80.5%)	0.116	0.67 (0.41–1.09)	0.100
Type of diabetes	1360				0.0195		
T1DM		28 (2.1%)	23/999 (2.3%)	5/361 (1.4%)		0.58 (0.19–1.42)	0.274
T2DM		1277 (93.9%)	929/999 (92.9%)	348/361 96.4%)		Ref	Ref
GDM		20 (1.5%)	20/999 (2%)	0/361 (0%)		(NA –76.9)	0.946
MODY		3 (0.2%)	2/999 (0.2%)	1/361 (0.3%)		0.74 (0.30–1.66)	0.501
Others		32 (2.3%)	25/999 (25%)	7/361 (1.9%)		1.33 (0.06–14.00)	0.814
HbA1c (%)^*∗*^	459	7.2 (6.5–8.5)	7.2 (6.4–8.4)	7.6 (6.7–8.7)	0.120	1.07 (0.94–1.21)	0.271
Hypertension (*N* (%))^*∗*^	1364	1131 (82.9%)	827/1003 (82.5%)	304/361 (84.2%)	0.447	1.14 (0.82–1.58)	0.447
Hyperlipidemia (*N* (%))	1364	459 (33.7%)	353/1003 (35.2%)	106/361 (29.4%)	0.044	0.77 (0.59–0.99)	0.045
Heart failure (*N* (%))^*∗*^	1364	235 (17.2%)	129/1003 (12.9%)	106/361 (29.4%)	<0.001	2.82 (2.10–3.77)	<0.001
Coronary artery disease (*N* (%))^*∗*^	1364	394 (28.9%)	263/1003 (26.2%)	131/361 (36.3%)	<0.001	1.60 (1.24–2.07)	<0.001
History of myocardial infarction (*N* (%))^*∗*^	1364	228 (16.7%)	146/1003 (14.6%)	82/361 (22.7%)	<0.001	1.73 (1.27–2.33)	<0.001
Atrial fibrillation (*N* (%))^*∗*^	1364	253 (18.5%)	159/1003 (15.9%)	94/361 (26%)	<0.001	1.87 (1.40–2.49)	<0.001
History of stroke (*N* (%))^*∗*^	1364	157 (11.5%)	106/1003 (10.6%)	51/361 (14.1%)	0.069	1.39 (0.97–1.98)	0.070
COPD (*N* (%))^*∗*^	1364	115 (8.4%)	68/1003 (6.8%)	47/361 (13%)	<0.001	2.06 (1.38–3.04)	<0.001
CKD (*N* (%))^*∗*^	1364	202 (14.8%)	119/1003 (11.6%)	83/361 (23%)	<0.001	2.22 (1.62–3.02)	<0.001
History of neoplasm (*N* (%))^*∗*^	1364	163 (12%)	116/1003 (11.6%)	47/361 (13%)	0.465	1.14 (0.79–1.63)	0.465
Prehospital diabetes treatment	1312						
Metformin (*N* (%))^*∗*^		697 (53.1%)	559/985 (56.8%)	138/327 (42.2%)	<0.001	0.56 (0.43–0.72)	<0.001
SU (*N* (%))		242 (18.4%)	181/985 (18.4%)	61/327 (18.7%)	0.910	1.02 (0.73–1.4)	0.910
DPP4-I (*N* (%))		27 (2.1%)	19/985 (1.9%)	8/327 (2.4%)	0.568	1.28 (0.52–2.84)	0.569
GLP-1RA (*N* (%))		5 (0.4%)	4/985 (0.4%)	1/327 (0.3%)	1	0.75 (0.04–5.11)	0.799
SGLT2i (*N* (%))		76 (5.8%)	60/985 (6.1%)	16/327 (4.9%)	0.422	0.79 (0.44–1.36)	0.422
Insulin (*N* (%))^*∗*^		467 (35.6%)	323/985 (32.8%)	144/327 (44%)	<0.001	1.61 (1.25–2.08)	<0.001
In-hospital diabetes treatment	1297						
Metformin (*N* (%))		457 (35.2%)	433/977 (44.3%)	24/320 (7.5%)	<0.001	0.10 (0.06–0.15)	<0.001
Sulfonylureas (*N* (%))		161 (12.4%)	141/977 14.4 (%)	20/320 (6.3%)	<0.001	0.40 (0.24–0.63)	<0.001
DPP4 inhibitors (*N* (%))		19 (1.5%)	18/977 (1.8%)	1/320 (0.3%)	0.058	0.17 (0.01–0.81)	0.082
GLP-1 receptor agonists (*N* (%))		6 (0.5%)	6/977 0.6 (%)	0/320 (0%)	0.346	0.0 (0->1000)	0.97
SGLT2 inhibitors (*N* (%))		84 (6.5%)	80/977 (8.2%)	4/320 (1.3%)	<0.001	0.14 (0.04–0.34)	<0.001
Insulin (*N* (%))		800 (61.7%)	563/977 (57.6%)	237/320 74.1 (%)	<0.001	2.1 (1.59–2.79)	<0.001
In-hospital treatment	1364						
Antiplatelet drugs (*N* (%))^*∗*^		254 (18.6%)	193/1003 (19.2%)	61/361 (16.9%)	0.326	0.85 (0.62–1.17)	0.327
VKA/NOAC (*N* (%))		90 (6.6%)	81/1003 (8.1%)	9/361 (2.5%)	<0.001	0.29 (0.13–0.6	<0.001
ACEI/ARB (*N* (%))^*∗*^		274 (20.1%)	233/1003 (23.2%)	41/361 (11.4%)	<0.001	0.42 (0.29–0.60)	<0.001
Beta-blocker (*N* (%))^*∗*^		433 (31.7%)	339/1003 (33.8%)	94/361 (26%)	0.007	0.69 (0.53–0.90)	<0.001
CCB (*N* (%))^*∗*^		219 (16.1%)	190/1003 (18.9%)	29/361 (8%)	<0.001	0.37 (0.24–0.56)	<0.001
Loop diuretics (*N* (%))^*∗*^		531 (38.9%)	326 (32.5%)	205 (56.8%)	<0.001	2.73 (2.13–3.50)	<0.001
Thiazide diuretics (*N* (%))^*∗*^		120 (8.8%)	113/1003 (11.3%)	7/361 (1.9%)	<0.001	0.16 (0.07–0.31)	<0.001
Statin (*N* (%))^*∗*^		237 (17.4%)	191/1003 (19%)	46/361 (12.7%)	0.007	0.62 (0.43–0.87)	0.007
Remdesivir (*N* (%))		98 (7.2%)	75/1003 (7.5%)	23/361 (6.4%)	0.485	0.84 (0.51–1.34)	0.486
Dexamethasone (*N* (%))		353 (25.9%)	213/1003 (21.2%)	140/361 (38.8%)	<0.001	2.35 (1.81–3.05)	<0.001
LMWH (*N* (%))		675 (49.5%)	465/1003 (46.4%)	210/361 (58.2%)	<0.001	1.61 (1.26–2.05)	<0.001
On-admission presentation							
SBP (mmHg)	1181	131 (116–147)	133 (120–149)	125 (100–140)	<0.001	0.99 (0.98–0.99)	<0.001
DBP (mmHg)	1181	79 (69–87)	80 (70–88)	72 (60–82)	<0.001	0.97 (0.96–0.98)	<0.001
Heart rate (bpm)	1196	82 (74–95)	82 (74–94)	82 (73–100)	0.367	1.00 (1.00–1.01)	0.270
Respiratory rate (*N*)	947	16 (12–20)	15 (12–18)	18 (14–22)	<0.001	1.10 (1.07–1.13)	<0.001
Oxygen saturation (%)	1109	95 (91–97)	95 (91–97)	93 (88–96)	<0.001	0.96 (0.94–0.97)	<0.001
On-admission laboratory results							
Plasma glucose (mmol/L)^*∗*^	1127	8.1 (6.0–11.5)	7.78 (5.9–10.83)	9 (6.32–13.5)	0.002	1.04 (1.01–1.06)	0.001
Plasma glucose >10 mmol/l (*N* (%))^*∗*^	1127	366 (32.5%)	251/852 (29.5%)	115/275 (41.8%)	<0.001	1.72 (1.30–2.28)	<0.001
Creatinine (mmol/l)^*∗*^	942	87.2 (66.4–132.8)	81 (63.98–109)	122.5 (78.95–188)	<0.001	1.00 (1.00–1.00)	<0.001
CRP (mg/l)^*∗*^	1321	58.1 (23.4–109)	49.6 (18.7–90.3)	93.4 (46.6–165)	<0.001	1.01 (1.01–1.01)	<0.001
D-dimer (*μ*g/ml)^*∗*^	1259	1.1 (0.6–2.3)	0.92 (0.54–1.78)	1.69 (0.94–4,42)	<0.001	1.07 (1.05–1.09)	<0.001
WBC (10^3^/mm^3^)^*∗*^	1319	6.6 (4.8–9.5)	6.29 (4.62–8.67)	8.25 (5.62–12.61)	<0.001	1.11 (1.08–1.14)	<0.001

^Data are presented as median (Q1-Q3); ^*∗*^variable used in logistic regression modeling. BMI, body mass index; T1DM, type 1 diabetes mellitus; T2DM, type 2 diabetes mellitus; MODY, maturity onset diabetes of the young; GDM, gestational diabetes mellitus; COPD, chronic obstructive pulmonary disease; CKD, chronic kidney disease; SU, sulphonylurea; GLP-1, glucagon-like peptide-1 agonists; DPP4, dipeptidyl peptidase-4 inhibitors; SGLT2, sodium-glucose co-transporter-2 inhibitors; VKA, vitamin K antagonists; NOAC, new oral anticoagulant; ACEI, angiotensin-converting enzyme inhibitors; ARB, angiotensin II receptor blockers; CCB, calcium-channel blockers; LMWH, low-molecular weight heparin; SBP, systolic blood pressure; DBP, diastolic blood pressure; bpm, beats per minute; CRP, C-reactive protein; WBC, white blood count; HbA1c, glycated hemoglobin.

**Table 3:** Multivariable logistic regression analysis for the risk of in-hospital death in DP.

Variable	OR	95% CI	*P* value
Age >65 years (yes/no)	3.02	2.02–4.51	<0.001
Heart failure (yes/no)	1.80	1.16–2.80	0.009
Coronary artery disease (yes/no)	1.03	0.64–1.66	0.890
Atrial fibrillation (yes/no)	1.00	0.65–1.53	0.993
COPD (yes/no)	1.30	0.76–2.21	0.339
CKD (yes/no)	1.67	1.06–2.62	0.027
History of myocardial infarction (yes/no)	1.18	0.70–1.99	0.546
Prehospital metformin use (yes/no)	0.73	0.51–1.05	0.091
Prehospital insulin use (yes/no)	1.44	1.01–2.07	0.046
CRP on admission (mg/l)	1.01	1.00–1.01	<0.001
D-dimer on admission (*μ*g/ml)	1.04	1.02–1.07	0.001
Glycemia on admission over 10 mmol/L (yes/no)	1.42	1.01–2.01	0.048
WBC (10^3^/mm^3^)	1.03	0.99–1.06	0.074
ACEI/ARB use (yes/no)	0.82	0.48–1.40	0.460
Beta blocker use (yes/no)	0.98	0.63–1.53	0.924
CCB use (yes/no)	0.37	0.19–0.69	0.002
Loop diuretics use (yes/no)	2.14	1.48–3.10	<0.001
Thiazide diuretics use (yes/no)	0.31	0.12–0.86	0.024
Statin use (yes/no)	0.48	0.28–0.83	0.008

COPD, chronic obstructive pulmonary disease; ACEI, angiotensin-converting enzyme inhibitors; ARB, angiotensin II receptor blockers; CCB, calcium-channel blockers; CRP, C-reactive protein; WBC, white blood count; Nagelkerke's *R*^2^ = 30.9%.

**Table 4:** Uni- and multivariate analyses for the risk of stay in the intensive care unit (ICU) in DP.

Variable	Univariate logistic regression			Multivariate logistic regression		
	OR	95% CI	*P* value	OR	95% CI	*P* value
Gender (female)	0.62	0.45–0.83	0.002	0.92	0.58–1.45	0.718
Age (years)	0.98	0.97–1	0.006	0.96	0.94–0.99	0.001
Age >65 years (yes/no)	0.8	0.6–1.08	0.149			
BMI (kg/m^2^)^∧^	1.03	1.0–1.06	0.044			
BMI >25 kg/m^2^ (yes/no)	1.57	0.88–2.99	0.146			
Hypertension (yes/no)	0.91	0.63–1.35	0.63			
History of stroke (yes/no)	0.67	0.39–1.09	0.124			
HF (yes/no)	0.79	0.51–1.17	0.248			
Coronary artery disease (yes/no)	0.95	0.68–1.31	0.766			
Atrial fibrillation (yes/no)	0.65	0.42–0.97	0.042	0.616	0.34–1.13	0.116
COPD (yes/no)	0.93	0.52–1.55	0.780			
CKD (yes/no)	1.06	0.7–1.57	0.784			
History of myocardial infarction (yes/no)	0.82	0.54–1.22	0.342			
History of neoplasm (yes/no)	0.73	0.43–1.16	0.202			
Prehospital metformin use (yes/no)	0.81	0.6–1.11	0.187			
Prehospital insulin use (yes/no)	1.29	0.93–1.76	0.120			
HbA1c (%)	1.11	0.95–1.28	0.153			
CRP on admission (mg/l)	1.01	1.01–1.01	<0.001	1.01	1.00–1.01	<0.001
D-dimer on admission (*μ*g/ml)	1.04	1.02–1.06	<0.001	1.01	0.98–1.05	0.562
Creatinine on admission (mmol/l)	1.00	1.00–1.00	0.60			
Glycemia on admission (mmol/L)	1.05	1.02–1.07	<0.001			
Glycemia on admission over 10 mmol/L (yes/no)	2.32	1.64–3.29	<0.001	1.87	1.20–2.92	0.006
WBC (10^3^/mm^3^)	1.01	1.0–1.02	0.046	1.02	0.96–1.05	0.307
Antiplatelet drugs use (yes/no)	1.7	1.2–2.39	0.002	0.98	0.58–1.70	0.939
ACEI/ARB use (yes/no)	0.81	0.54–1.17	0.267			
Beta-blocker use (yes/no)	1.45	1.07–1.96	0.016	0.79	0.49–1.27	0.332
CCB use (yes/no)	1.16	0.78–1.68	0.461			
Loop diuretics use (yes/no)	30.36	18.53–53.4	<0.001	47.92	23.34–98.41	<0.001
Thiazide diuretics use (yes/no)	0.41	0.19–0.78	0.012	0.30	0.11–0.86	0.025
Statin use (yes/no)	0.99	0.67–1.45	0.971			

^BMI variable was not used in multivariable analysis due to many data missing; BMI, body mass index; HF, heart failure; COPD, chronic obstructive pulmonary disease; CKD, chronic kidney disease; ACEI, angiotensin-converting enzyme inhibitors; ARB, angiotensin II receptor blockers; CCB, calcium-channel blockers; CRP, C-reactive protein; WBC, white blood count; HbA1c, glycated hemoglobin; Nagelkerke's *R*^2^ = 46.4%.

## Data Availability

The data used to support the findings of this study are available from the corresponding author upon request.

## Supplementary Materials

Supplementary Materials Table S1: Comparison of DP according to in-hospital metformin discontinuation status.
