# Supplementary material for: Characteristics, Mortality, and Clinical Outcomes of Hospitalized Patients with COVID-19 and Diabetes: A Reference Single-Center Cohort Study from Poland
Source: Int J Endocrinol. 2023 Feb 16;2023:8700302. doi: 10.1155/2023/8700302 (PMC9949948; doi:10.1155/2023/8700302)
Supplement: Supplementary Materials — Table S1: Comparison of DP according to in-hospital metformin discontinuation status. [file 8700302.f1.docx]

| **Table S1** Comparison of DP according to in-hospital metformin discontinuation status. | | | | | |
| --- | --- | --- | --- | --- | --- |
| **Characteristics** | **Available data** |  | **Metformin discontinued** | **Metformin sustained** | **P value** |
| Number | 666 |  | 283 | 383 | - |
| Age [years] | 666 |  | 70 (62-76) | 69 (62-75) | 0.344 |
| Age class >65 years [N(%)] | 666 |  | 176 (62.2%) | 240 (62.7%) | 0.901 |
| BMI [kg/m^2^] | 294 |  | 31.0 (27.3-34.8) | 29.7 (26.6-35.4) | 0.593 |
| Hypertension [N(%)] | 666 |  | 248 (87.6%) | 324 (84.6%) | 0.266 |
| Heart failure [N(%)] | 666 |  | 41 (14.5%) | 37 (9.7%) | 0.055 |
| Coronary artery disease [N(%)] |  |  | 86 (30.4%) | 96 (25.1%) | 0.128 |
| Oxygen saturation [%] on admission | 566 |  | 94 (89.3-96.8) | 95 (92-97) | 0.028 |
| Plasma glucose [mmol/L] on admission | 594 |  | 8.2 (6.3-12.0) | 8.1 (6-10.4) | 0.05 |
| CRP [mg/l] on admission | 651 |  | 74 (31.4-145) | 48.6 (16.1-95.0) | <0.001 |
| D-dimer [µg/ml] on admission | 630 |  | 1.1 (0.58-1.9) | 0.8 (0.5-1.7) | 0.011 |
| **Endpoints** |  |  |  |  |  |
| In-hospital death [Yes (%)] | 666 |  | 99 (35.0%) | 21 (5.5%) | <0.001 |
| Mechanical ventilation  [Yes (%)] | 666 |  | 61 (21.6%) | 25 (6.5%) | <0.001 |
| Admission to an ICU [n (%)] | 666 |  | 56 (19.8%) | 26 (6.8%) | <0.001 |
| data are presented as median (Q1-Q3) or N[%]; BMI – body mass index; CRP – C reactive protein; ICU – intensive care unit | | | | | |
